# The Role of Prophylactic Central Neck Dissection in the Treatment of Differentiated Thyroid Cancer

**DOI:** 10.5041/RMMJ.10234

**Published:** 2016-01-28

**Authors:** Max Hennessy, David Goldenberg

**Affiliations:** Department of Surgery, Division of Otolaryngology-Head and Neck Surgery, The Pennsylvania State University, College of Medicine, Hershey, PA, USA

**Keywords:** Differentiated thyroid cancer, morbidity, prophylactic central neck dissection, recurrence, survival, treatment

## Abstract

The utility and efficacy of prophylactic central neck dissection with total thyroidectomy for the treatment of differentiated thyroid cancer has been debated in the literature over the past few decades. Proponents of prophylactic central neck dissection support its routine use with the notion that it reduces local recurrence, increases accuracy in TNM staging, and reduces surgical morbidity associated with reoperation. Conversely, those against the use of routine prophylactic central neck dissection argue there is no clear evidence which shows a reduction in recurrence or added benefit to survival, while the procedure increases the risk for complications and morbidity. This article discusses the role of prophylactic central neck dissection in the setting of thyroid cancer and reviews recently published literature to evaluate efficacy and safety of this procedure.

## INTRODUCTION

Thyroid cancer is the most common endocrine malignancy, and its incidence is increasing at the highest rate among cancers in both the US and worldwide.^1^,^2^ The National Cancer Institute’s annual Surveillance Epidemiology and End Results (SEER) database estimates that there will be 62,450 new cases of thyroid cancer in the US in 2015, with an incidence of 13.5 per 100,000.^1^ The absolute increase in the incidence of thyroid cancer is estimated to be 9.4 per 100,000 individuals, with papillary thyroid cancer (PTC) accounting for the majority of these cases. Overall, differentiated thyroid cancer (DTC) has a 10-year survival rate of greater than 90%. However, despite its promising survival rate, local recurrence occurs in 20%–30% of papillary thyroid cancer patients due to clinically undetectable metastasis to cervical lymph nodes.^3^ Cervical lymph node metastases are a common feature of PTC, occurring primarily in the central compartment (level VI) with an incidence between 20% and 90% (average 60%).^4^–^8^ Conversely, follicular thyroid cancer (FTC) often spreads hematogenously, and rarely metastasizes to the cervical lymph nodes.^9^ Hurthle cell thyroid cancer (HTC) is a rare and aggressive form of differentiated thyroid cancer of follicular cell origin; HTC displays a lower rate of cervical lymph node metastasis compared to PTC.^9^

The central compartment is bounded by the hyoid bone (superior), carotid artery (lateral), and sternal notch or innominate artery (inferior). The American Thyroid Association (ATA) defines central compartment neck dissection as “comprehensive, compartment-oriented removal of the prelaryngeal and pretracheal nodes and at least one paratracheal lymph node basin.”^10^ The regional metastases to the cervical lymph nodes were traditionally believed to have an effect only on recurrence rate, but not mortality.^5^,^8^,^11^ However, in 2006, a population-based study from Sweden found lymph node metastases in both the central and lateral compartments to be a prognostic factor for patients with DTC.^11^ This finding complicated debate in the literature with regard to the initial treatment of differentiated thyroid cancer.^5^,^8^,^11^ Surgery, typically in the form of a total thyroidectomy (TT), has been accepted as the treatment of choice for most patients with differentiated thyroid cancer. There is also consensus in regard to treating patients with clinically evident level VI nodal disease with central neck dissection at the time of initial surgery.^4^–^8^ However, the addition of a prophylactic central neck dissection (PCND) to TT in clinically node-negative patients with DTC remains controversial due to lack of prospective randomized controlled studies.^4^,^12^ The ATA addresses this controversy in the 2015 American Thyroid Association Guidelines for Adult Patients with Thyroid Nodules and DTC, recommending the following for the use of PCND in the treatment of DTC:

(b) Prophylactic central-compartment neck dissection (ipsilateral or bilateral) should be considered in patients with papillary thyroid carcinoma with clinically uninvolved central neck lymph nodes (cN0) who have advanced primary tumors (T3 or T4), clinically involved lateral neck nodes (cN1b), or if the information will be used to plan further steps in therapy. (Weak Recommendation, Low-quality evidence)
(c) Thyroidectomy without prophylactic central neck dissection may be appropriate for small (T1 or T2), noninvasive, clinically node-negative PTC (cN0) and most follicular cancers. (Strong Recommendation, Moderate-quality evidence). (Recommendation 36, page 88)^4^

Thus, these recommendations do not definitively state when to complete a PCND. The inability definitively to state when to perform a PCND can be largely attributed to the fact that most of the published studies on PCND are retrospective. Thus the ability to assess the efficacy of PCND in improving long-term patient morbidity and survival is greatly diminished.^12^ To address this issue, a subcommittee of the ATA Surgical Affairs Committee attempted to determine the feasibility of a prospective randomized controlled trial of TT with PCND versus TT alone without PCND for clinically node-negative patients with PTC. The ATA subcommittee found that after surgery the low rates of morbidity and newly identified structural disease in clinically node-negative patients cause the sample size and length of follow-up required for a statistically significant outcome to become too large for the study to be feasible.^12^ Thus, until such a study is accomplished, the decision of when to perform a PCND must be based on retrospective studies and expert opinion.

## DISCUSSION

Proponents of routine PCND argue that because the central compartment is the major site for nodal metastasis in DTC, prophylactically removing lymph nodes will decrease the rate of recurrence from microscopic occult nodal metastases.^3^,^4^–^8^,^13^ The proposed benefits of PCND are reduction in local recurrence and increased accuracy in TNM staging which assists with subsequent treatment. This is in part due to the fact that identification of micrometastasis “upstages” PTC from Nx to N1a disease in the American Joint Committee on Cancer (AJCC) staging system, and N1a PTC is considered “stage III” PTC in patients >45 years.^4^ Additionally, a PCND should promote reduction in surgical morbidity associated with reoperation.^6^,^8^,^13^ Conversely, those against the use of routine PCND argue that there is no clear evidence which shows a reduction in recurrence or mortality, it increases operative time, and it increases risk for complications and short-term morbidity.^3^,^6^,^13^ Since the publication of the ATA’s recommendations for PCND in the management of DTC, there has been a great deal of research published on the topic. Most notably, five different meta-analyses have been published on the effects and outcomes of PCND.^6^–^8^,^14^,^15^ These studies provide valuable information on the effects of PCND on local recurrence, morbidity and complications, and subsequent treatment of DTC.

### Effects on Survival

With the 10-year survival rate for DTC being greater than 90%, the effect of PCND on survival is extremely difficult to determine due to the length of follow-up required and other causes of death that occur in the follow-up period. For this reason, there are very few studies in the literature which examine the effect of PCND on survival, with most studies opting to examine the effect of PCND on local recurrence and surgical morbidity. One retrospective study by Barczynski et al. did analyze 10-year disease-specific survival for patients with PTC who underwent TT alone versus TT with PCND. They examined 640 patients and found that those who underwent TT alone and TT with PCND had 10-year survival rates of 92.5% and 98%, respectively.^16^ This difference was statistically significant and suggests that PCND may improve long-term survival. However, the patients who received a PCND were also more than twice as likely to receive radioactive iodine ablation (RAI) therapy, and thus the data do not definitively conclude that PCND improves survival. As mentioned before, no prospective randomized controlled trials exist which examine the effect of PCND.

### Effects on Local Recurrence

Since the central compartment is a common site of recurrence, the basis of a PCND is to reduce the risk of local recurrence both by removing potential metastatic sites and by providing lymph nodes for histological analysis to identify micrometastasis.^4^,^17^ Since 2010, four meta-analyses were published that investigated the effect of TT with PCND on local recurrence rates in comparison to TT alone. None of these studies was able definitively to conclude that TT with PCND significantly decreased the risk of local recurrence.^6^–^8^,^14^ Three of the studies were able to demonstrate a trend of reduced local recurrence in TT with PCND versus TT alone; however, they were unable to show statistical significance.^6^,^8^,^14^ Lang et al. were able to show a statistically significant reduction of 35% in local recurrence for the TT with PCND group in comparison to TT alone. However, in their study, the TT with PCND patient group was also shown to be more likely to receive RAI therapy than the TT alone patient group. This difference was found to be statistically significant, and the authors were unable to conclude that the reduction in local recurrence was due to the PCND and not the increased use of RAI.^7^ In this study, the reduction was shown for short-term follow-up (<5 years), and due to the indolent nature of DTC a longer follow-up is needed to examine the long-term recurrence rates.^12^ The retrospective study by Barczynski et al., however, was able to show a significant reduction in the 10-year local recurrence rate of TT with PCND versus TT alone in the absence of RAI. They showed in patients who did not receive RAI therapy that PCND with TT had a 10-year local recurrence rate of 3.9% compared to the TT alone rate of 14.8%.This statistically significant difference was lost when TT with PCND and TT alone were compared in the presence of RAI therapy following surgery.^16^

In a retrospective study by Moreno et al., the absence of macroscopic nodal metastasis on preoperative ultrasound of the central compartment was found to be to be a predictor of recurrence-free survival in patients with PTC.^18^ They examined the effect of histological analysis of lymph nodes obtained from PCND on 10-year recurrence-free survival. The data obtained showed that the 10-year recurrence-free survival in patients with micrometastasis identified after PCND compared to node-negative patients after PCND was not statistically significant.^18^ Thus, the identification of macroscopic nodal metastasis in the central compartment on ultrasound is a significant prognostic factor in DTC, whereas micrometastasis identified pathologically in patients after PCND is not. In a review, Steward and colleagues also assert that the discovery of micrometastasis in the central neck compartment does not alter 10-year-survival and thus discovery of these is not sufficient to justify a PCND.^13^ With the identification of microscopic nodal metastasis occurring frequently, the identification must be used carefully when staging a patient.^4^ Upstaging a patient from N0 to N1a upgrades a patient >45 years old from stage I to stage III on the AJCC staging system. This is problematic, as microscopic nodal metastases do not display the same risk for recurrence as macroscopic nodal metastases and can cause an unnecessary increase in radioactive iodine utilization.^4^ Thus, PCND should not be used with the intention of reducing recurrence and improving staging by identifying and removing micrometastases.

### Morbidity of Central Neck Dissection

An important area for consideration in regard to PCND is the associated risks, which is why the ATA stated with Recommendation 36 that its recommendations should be considered in light of surgical expertise. There is increased cost and morbidity in patients with recurrent disease, given that reoperative cervical surgery is associated with higher risk of recurrent laryngeal nerve injury and hypoparathyroidism, both transient and permanent.^19^,^20^ This is due in part to the more extensive dissection of the central compartment, which is more likely to damage the recurrent laryngeal nerve and blood supply to the parathyroids in comparison to TT alone.^1^,^6^,^14^ Four meta-analyses on PCND in the treatment of DTC have been published that address the complications of PCND and its effects on morbidity. A fifth meta-analysis by Zetoune et al. examined the morbidity associated with the addition of PCND to TT; however, only two of the studies included in their analysis examined the associated complications, and, as such, this study does not carry as much weight as the four others.^8^ In these five meta-analyses, the only significant complication found with TT with PCND compared to TT alone was temporary hypoparathyroidism.^6^–^8^,^14^,^15^ The risks of permanent hypoparathyroidism, and temporary and permanent recurrent laryngeal nerve damage, were not found to be different between the two groups. Nonetheless, it does come with an increased risk of temporary hypoparathyroidism and, as such, should be performed in the hands of an experienced surgeon.^19^

With the knowledge that PCND is relatively safe with rare long-term morbidities, the argument that PCND will prevent the potential morbidity associated with reoperation seems attractive. However, because PCND has not been shown to reduce local recurrence rates, the argument for routine PCND to prevent reoperation is problematic, as a reoperation is only necessary in the setting of disease recurrence.

## CONCLUSION

The use of routine prophylactic central neck dissection for the treatment of differentiated thyroid cancer has been an area of debate over the past few decades. With the lack of prospective randomized controlled trials in the literature and impractical requirements needed to accomplish such a trial, the decision of when to perform a prophylactic central neck dissection must be based on retrospective studies and expert opinion. Based on current literature, we support the American Thyroid Association’s recommendations that prophylactic central neck dissection should be reserved only for the use in invasive or advanced (T3 and T4) papillary thyroid cancers. Papillary thyroid carcinomas that are small and non-invasive, as well as most follicular thyroid carcinomas, should be treated with thyroidectomy alone due to the very low rates of recurrence in these patient groups. When planning the initial treatment of a patient with differentiated thyroid cancer one must determine the efficacy of prophylactic central neck dissection. In conclusion, the benefits of prophylactic central neck dissection are too meager to outweigh its associated risks, and thus the use of central neck dissection should be reserved for high-risk patients in the hands of an experienced surgeon.

## Abbreviations:

ATA: American Thyroid Association
DTC: differentiated thyroid cancer
FTC: follicular thyroid cancer
HTC: Hurthle cell carcinoma
PCND: prophylactic central neck dissection
PTC: papillary thyroid cancer
RAI: radioactive iodine ablation
SEER: Surveillance Epidemiology and End Results
TT: total thyroidectomy.
